# Chloroplast genomes of *Caragana tibetica* and *Caragana turkestanica*: structures and comparative analysis

**DOI:** 10.1186/s12870-024-04979-9

**Published:** 2024-04-09

**Authors:** LiE Liu, HongYan Li, JiaXin Li, XinJuan Li, Na Hu, Jing Sun, Wu Zhou

**Affiliations:** 1 College of Eco-Environmental Engineering, Qinghai University, Xining, 810016 China; 2 Qinghai Provincial Key Laboratory of Qinghai-Tibet Plateau Biological Resources, Northwest Institute of Plateau Biology, Chinese Academy of Sciences, Xining, 810008 China

**Keywords:** *Caragana*, Chloroplast DNA, Phylogenomics, Species divergence, Species identification

## Abstract

**Background:**

The genus *Caragana* encompasses multiple plant species that possess medicinal and ecological value. However, some species of *Caragana* are quite similar in morphology, so identifying species in this genus based on their morphological characteristics is considerably complex. In our research, illumina paired-end sequencing was employed to investigate the genetic organization and structure of *Caragana tibetica* and *Caragana turkestanica*, including the previously published chloroplast genome sequence of 7 *Caragana* plants.

**Results:**

The lengths of *C. tibetica* and *C. turkestanica* chloroplast genomes were 128,433 bp and 129,453 bp, respectively. The absence of inverted repeat sequences in these two species categorizes them under the inverted repeat loss clade (IRLC). They encode 110 and 111 genes (4 /4 rRNA genes, 30 /31tRNA genes, and 76 /76 protein-coding genes), respectively. Comparison of the chloroplast genomes of *C. tibetica* and *C. turkestanica* with 7 other *Caragana* species revealed a high overall sequence similarity. However, some divergence was observed between certain intergenic regions (*matK-rbcL*, *psbD-psbM*, *atpA-psbI*, and etc.). Nucleotide diversity (π) analysis revealed the detection of five highly likely variable regions, namely *rps2-atpI*, *accD-psaI-ycf4*, *cemA-petA*, *psbN-psbH* and *rpoA-rps11*. Phylogenetic analysis revealed that *C. tibetica*’s sister species is *Caragana jubata*, whereas *C. turkestanica*’s closest relative is *Caragana arborescens*.

**Conclusions:**

The present study provides worthwhile information about the chloroplast genomes of *C. tibetica* and *C. turkestanica*, which aids in the identification and classification of *Caragana* species.

**Supplementary Information:**

The online version contains supplementary material available at 10.1186/s12870-024-04979-9.

## Background

*Caragana Fabr.* comprises over 100 species and belongs to the family of Fabaceae. These plants are mainly distributed in arid and semi-arid regions of Asia and Europe. Of these species, 66 were found in China, 32 of which are endemic [1]. *Caragana* plants are renowned for their drought, infertile conditions, cold and heat tolerance [2]. They are widely cultivated due to their ability to adapt to dry conditions [3]. Similar to other Fabaceae family plants, these plants can convert atmospheric nitrogen into usable nutrients via nodules on their roots, playing a role in rejuvenating infertile soils, combating dust storms, and hindering desertification [4]. The distribution of various *Caragana* plants in China has been extensively studied (Table 1).

**Table 1:** Introduction to 9 species of the genus *Caragana*

Species	Distribution in China	Habitat	Height	Notes
*Caragana arborescens Lam* [5, 6]	Northeastern, northern, northwestern regions	Dry slopes, grasslands, sandy areas, and hilly regions	4–5 m	Blooms in May; seeds mature in midsummer;Garden Ornamental and Greening Use; Medicinal value
*Caragana opulens Kom* [7]	Northern, northwestern, southwestern regions	Hilly areas up to 3400 m	0.4–0.6 m	Excellent green manure plant
*Caragana jubata (Pall.) Poir* [8]	Northern, southwestern regions	High mountain shrublands	0.3–2.0 m	The bark, stem, and leaves have the effects of connecting tendons and bones, dispelling wind and dampness, promoting blood circulation, removing swelling, and relieving pain.
*Caragana rosea Turcz. ex Maxim* [9]	Northeastern, northern, eastern, southern Gansu	Mountain slopes and valleys	0.4–1.0 m	Medicinal Value: clears heat and detoxifies
*Caragana kozlowii Kom* [10]	Lancang River and Tibet	Along rivers 3600-4000 m	0.5–1.5 m	Suitable for landscaping and cultivation in garden courtyards.
*Caragana microphylla Lam* [11]	Northeast, North, Northwest	Grows in fixed or semi-fixed sandy soil	1–3 m	Branches can be used as green manure; tender branches and leaves can be used as fodder. It is a plant for stabilizing sand and soil conservation.
*Caragana korshinskii Kom* [11]	Northeast, North, Northwest	Grows in fixed or semi-fixed sandy soil	1–4 m	Excellent sand-fixing and soil conservation plant.
*Caragana tibetica Kom* [12]	Western Inner Mongolia, Northern Shaanxi, Ningxia, Gansu, Qinghai, Western Sichuan, Tibet	Dry mountain slopes and sandy areas	0.2–0.3 m	Mentioned in Mongolian and Tibetan medicine.
*Caragana turkestanica Kom* [13]	Xinjiang: Jimunai, Habahe	Dry shrubs, sunny slopes	1–2 m	Suitable for landscaping and cultivation in garden courtyards.

The “notes” column indicates the species’ special contributions in terms of horticultural beautification or its medicinal properties

Additionally, previous studies have shown that this genus includes more than 10 plants with excellent pharmacological properties. These plants have been utilized to treat various diseases such as fever, inflammation, wound infections, headaches, rheumatoid arthritis, and cancer [4, 14, 15]. The *C. tibetica* studied in this article is mentioned in both Mongolian medicine and Tibetan medicine as a potential treatment for rheumatoid arthritis, wounds, hypertension, and anemia [4].

The current research indicates a limited availability of data on plants in *Caragana*, with only 14 chloroplast (cp) genomes reported*.* However, the evolutionary analyses using nuclear ITS (Internal Transcribed Spacer) and plastid marker sequence data (*matK*, *trnL-F*, and *psbA-trnH*) for studying the phylogenetic relationships of *Caragana* plants lack resolution, leaving unanswered questions about the classification of certain medicinal plants like *Caragana changduensis Liou f.*, *Caragana frutex (L.) C. Koch*, and *Caragana polourensis Franch.* [1, 16–19]. These plants, representing different species within *Caragana*, exhibit varying morphological features in terms of flowers, leaves, stems, and other aspects. Additionally, their habitat preferences contribute to morphological adaptations and variations, as they inhabit diverse ecological environments. Thus, relying solely on morphology for identifying different *Caragana* species may introduce errors and uncertainties. Consequently, finding an accurate and convenient method for plant identification in *Caragana* is crucial.

According to reports, researchers have gained a deeper understanding of chloroplasts, including their origins, structures, evolution, and genetic engineering [20, 21]. Chloroplasts contain their genetic system [22], and most plants have chloroplasts existing in the form of covalently closed circular DNA [23]. The rapid development of sequencing technologies has led to the discovery of more efficient molecular markers within the chloroplast genome, which are advantageous for accurate species identification. The chloroplast genome is an ideal choice for molecular identification, phylogenetic analysis, and species conservation research according to a previous study [24]. Unlike the nuclear genome, the chloroplast genome is particularly valuable for plant phylogenetic studies due to its unique features: it is typically inherited from only one parent, had a simpler structure, and contains multiple copies of each gene [23, 25]. The plastid chromosome, which is circular and has a length of 120 ~ 160kb [25], consists of four regions containing two inverted repeat regions (IRs). These regions separate the large single copy region (LSC) and the small single copy region (SSC) [26]. Due to their high level of conservation and relative small size, plastid structure and gene content is easy to obtain completely and worth studying in species identification, population genetics, and phylogenetics [27, 28]. Currently, various plants, such as Desmodieae, Picea, and Epimedium [29–31], utilize the chloroplast genome to study their phylogenetic relationships. Reports have indicated the occurrence of inverse repeated loss of the evolutionary branch (IRLC) in Fabaceae [32–36], including the 8 species of *Caragana* with IRLC that have been reported [1, 16, 17, 37]. Therefore, it will be possible to study *Caragana* as a lineage representing a broad spectrum of IRLC with the improvement in the chloroplast genome data of *Caragana*.

In this study, we compared the complete chloroplast genomes of *C. tibetica* and *C. turkestanica* to those of other species within the *Caragana* genus. Additionally, we analyzed the structural characteristics and phylogenetic relationships of these chloroplast genomes with other species within the Fabaceae family. The results of this study have advanced the knowledge of chloroplast genome data within the genus *Caragana*, providing valuable insights for species identification, systematic evolutionary studies, and germplasm conservation and utilization.

## Results

### Characteristics of *Caragana* chloroplast genomes

**Fig. 1:**
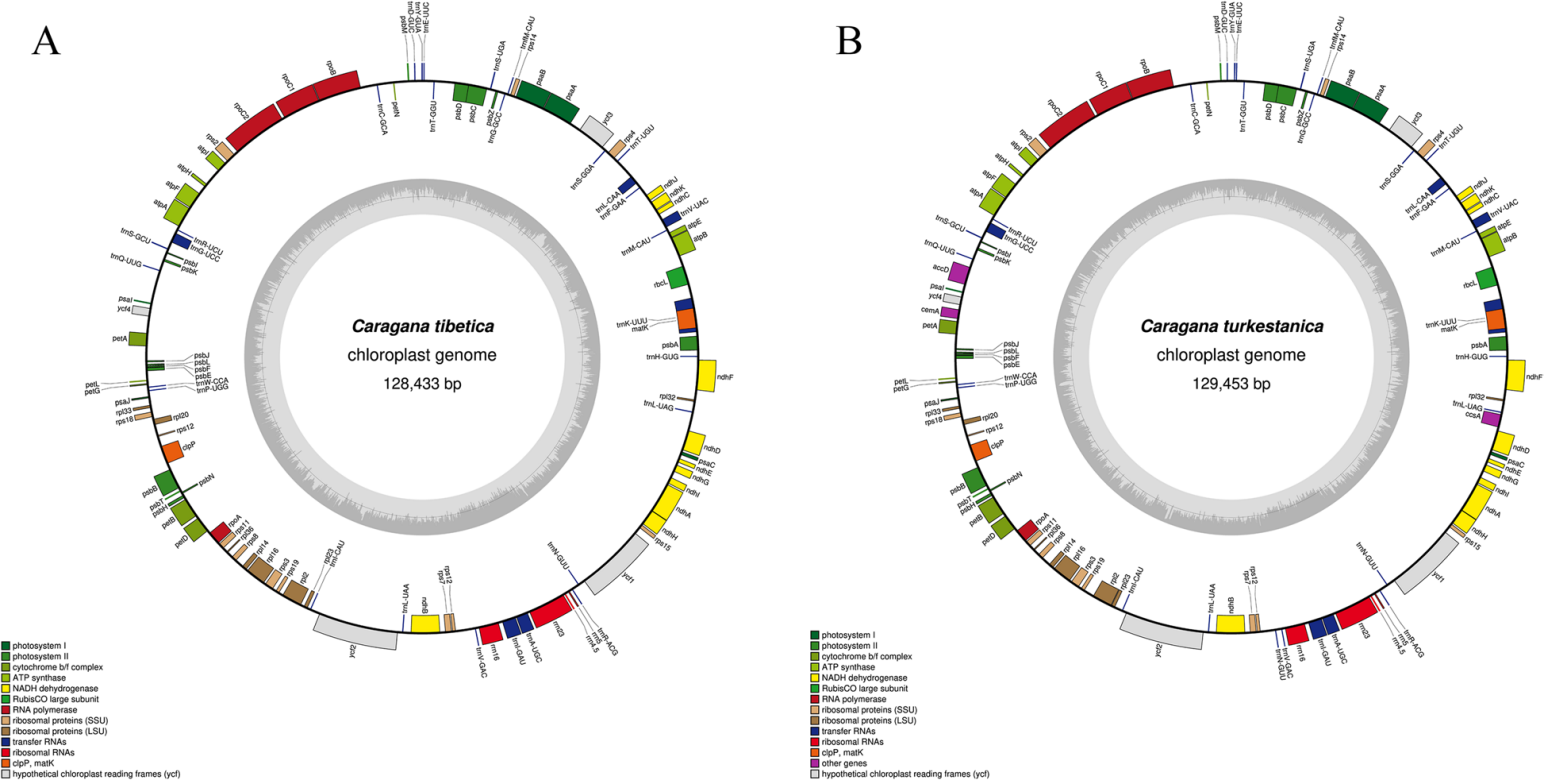
The diagram illustrates the chloroplast gene maps of *C. tibetica* (**A**) and *C. turkestanica* (**B**). The genes located on the outer circle are transcribed counterclockwise, whereas those on the inner circle are transcribed clockwise. Different functional gene groups are represented by different color codes. In addition, changes in GC content are represented by light gray in the inner circle, while changes in AT content are represented by dark gray

Draw gene maps using OGDRAW for *C. tibetica* (Fig. 1A) and *C. turkestanica* (Fig. 1B) based on the annotation results of their chloroplast genomes. The chloroplast genomes sizes of *C. tibetica* and *C. turkestanica* were found to be 128,433 and 129,453bp, respectively. With the loss of the IR region in the two plants, they do not have the typical quadripartite structure found in most flowering plants’ chloroplast genomes, and their lengths were accordingly shorter. Nevertheless, the cp genome structures, gene contents and direction were strongly comparable (Fig. 1A and B).

The annotation results of the chloroplast genomes revealed that *C. tibetica* had a total of 110 specific genes in chloroplast genome, while *C. turkestanica* shared totally 111 specific genes, comprising 76 protein coding genes, 31 (30) tRNA genes and 4 rRNA genes (Table 2). The GC contents of the two species were very similar, with values of 34.30% and 34.71%, respectively. Seven cp genomes in the *Caragana* species (*C. arborescens, C. opulens, C. jubata, C. rosea, C. microphylla, C. kozlowii, C. korshinskii*) with missing IR regions were compared with *C. tibetica* (128,433 bp) and *C. turkestanica* (129,453 bp). The results revealed that the total sequence lengths ranged from 128,132 to 133,122 base pairs. The deletion of the IR region resulted in the shortest chloroplast genome length of 128,132 bp in *C. jubata* and the longest in *C. rosea* (133,122 bp). In addition, the number of genes in *C. turkestanica*, *C. arborescens*, and *C. opulens* was one more than that in other species (tRNA encoded by trnN-GUU gene). At the same time, the number of protein-encoding genes and ribosomal RNA genes was consistent among the nine plants.

**Table 2:** Summary of complete chloroplast genomes for nine *Caragana* species

Plastome Characteristics		C. *tib*	*C. tur*	*C. arb*	*C. opu*	*C. jub*	*C. ros*	*C. mic*	*C. koz*	*C. kor*
GenBank accession		OQ942026	OQ942027	MT211962	OQ656872	MT211963	NC039932	NC032691	NC035228	NC035229
protein Coding gennes	Length(bp)	66,123	66,228	66,222	66,333	66,222	66,243	66,231	66,234	66,231
	GC(%)	37.01	36.88	36.89	37.01	36.91	37.13	36.88	37.03	36.88
	Length(%)	51.48	51.16	51.15	50	51.68	49.76	50.94	50.45	51.21
	Number	76	76	76	76	76	76	76	76	76
tRNA	Length(bp)	2,295	2,369	2,379	2,370	2,296	2,359	2,370	2,285	2,370
	GC(%)	52.59	52.72	52.74	52.83	52.87	52.73	53.14	53.15	53.05
	Length(%)	1.79	1.83	1.83	1.80	1.80	1.77	1.82	1.74	1.83
	Number	30	31	31	31	30	30	30	30	30
rRNA	Length(bp)	4,521	4,521	4,522	4,520	4,520	4,537	4,520	4,521	4,520
	GC(%)	54.88	54.81	54.8	54.56	54.76	54.77	54.82	54.75	54.82
	Length(%)	3.52	3.49	3.49	3.40	3.52	3.40	3.48	3.44	3.49
	Number	4	4	4	4	4	4	4	4	4
Total	Length(bp)	128,433	129,453	129,473	132,815	128,132	133,122	130,029	131,274	129,331
	Number Of genes	110	111	111	111	110	110	110	110	110
	GC(%)	34.65	34.30	34.30	34.71	34.42	34.84	34.26	34.50	34.36

Note: *C. tib* = *C. tibetica; C. tur* = *C. turkestanica; C. arb* = *C. arborescens; C. opu* = *C. opulens; C. jub* = *C. jubata; C. ros* = *C. rosea; C. mic* = *C. microphylla; C. koz* = *C. kozlowii; C. kor* = *C. korshinskii*

From the standpoint of gene contents, the nine plants had the highest number of protein-encoding genes, which accounted for approximately half of the entire genome length. Following the most abundant genes were tRNA genes, which were shorter in length than other genes. In general, the sequence length and gene content of chloroplast genomes in the nine *Caragana* species were roughly consistent. We also analyzed the difference in GC content among the three types of genes. The GC content of rRNA genes was highest, exceeding 50% and were consistently so. GC content of tRNA genes were next in amount. The lowest GC content was observed for protein-coding genes, which was approximately 37%. Moreover, the average GC content of the nine species were around 34%, which suggested that the sequence of *Caragana* species was relatively conserved during the process of evolution.

**Fig. 2:**
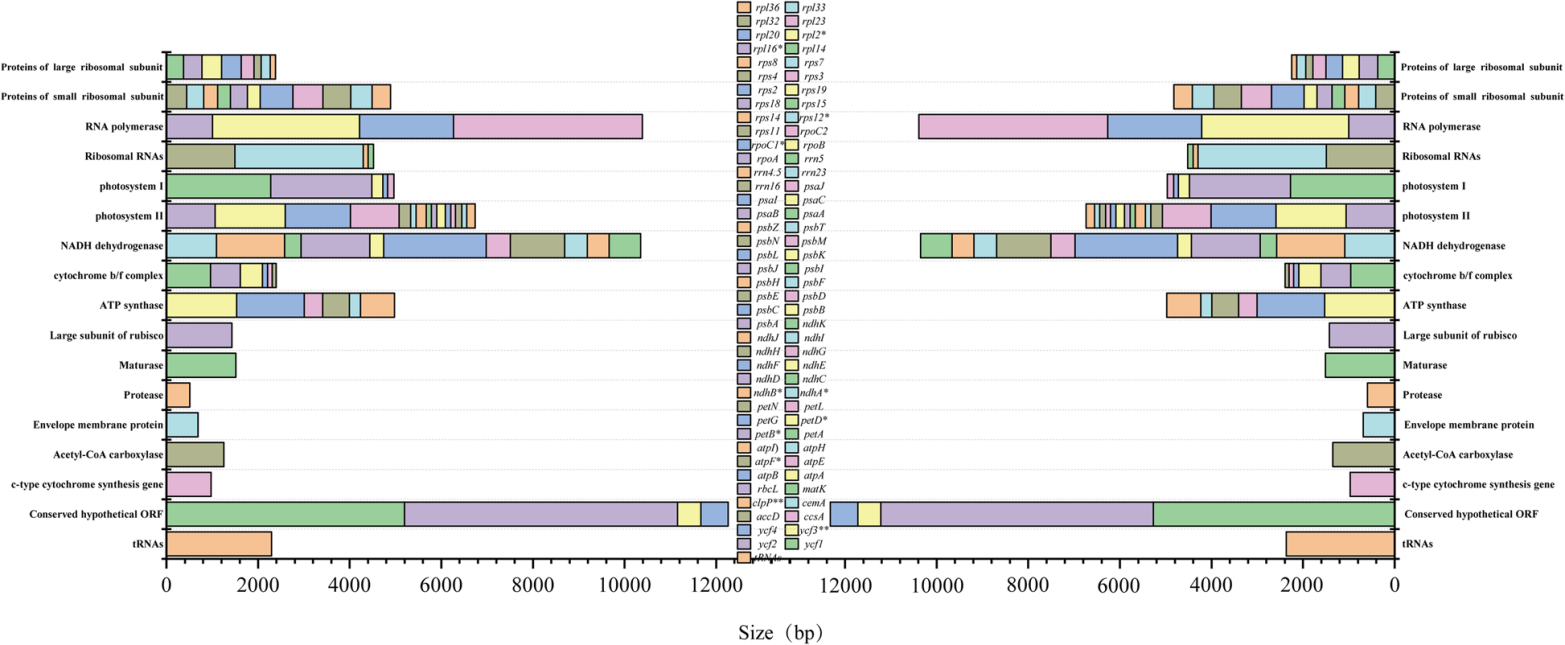
Gene contents of *C. tibetica* and *C. turkestanica* chloroplast genomes. The color of each gene is unique, and the horizontal axis indicates that each box is proportional to the size (bp) of the gene

The genes encoded by the chloroplast genomes of *C. tibetica* and *C. turkestanica* can be divided into three categories, similar to other species. There were 57 genes related to self-replication, including ribosomal RNA, transfer RNA, and three subunits (large, small, and DNA-dependent RNA polymerase) that encode chloroplast RNA polymerase; 44 photosynthesis-related genes; the remaining genes were categorized as other genes and unknown genes. In the chloroplast genomes of *C. tibetica* and *C. turkestanica*, 17 genes with introns were detected. Thereinto, *C. tibetica* had two genes (*clpP* and *ycf3*) with two introns, while *C. turkestanica* only had one gene (*ycf3*) with two introns, and the remaining 15 genes (*rpl16*, *rpl2*, *rps12*, *rpoC1*, *trnA-UGC*, *trnG-UCC*, *trnI-GAU*, *trnK-UUU*, *trnL-CAA*, *trnV-UAC*, *ndhA*, *ndhB*, *petB*, *petD*, *atpF*) had only one intron (Fig. 2, Table 3).

**Table 3:** Genes in the chloroplast genome of *Caragana* species

Category	Group of genes	Name of genes
Self-replication	Proteins of large ribosomal subunit	*rpl14*, rpl16*, rpl2*, *rpl20*, *rpl23*, *rpl32*, *rpl33*, *rpl36*
	Proteins of small ribosomal subunit	*rps11*, rps12*, *rps14*, *rps15*, *rps18*, *rps19*, *rps2*, *rps3*, *rps4*, *rps7*, *rps8*
	Subunits of RNA polymerase	*rpoA*, *rpoB*, rpoC1*, *rpoC2*
	Ribosomal RNAs	*rrn16*, *rrn23*, *rrn4.5*, *rrn5*
	Transfer RNAs	trnA-UGC*, *trnC-GCA*, *trnD-GUC*, *trnE-UUC*, *trnF-GAA*, *trnG-GCC*, trnG-UCC*, *trnH-GUG*, *trnI-CAU*, trnI-GAU*, trnK-UUU*, trnL-CAA*, *trnL-UAA*, *trnL-UAG*, *trnM-CAU*, *trnN-GUU*(2), *trnP-UGG*, *trnQ-UUG*, *trnR-ACG*, *trnR-UCU*, *trnS-GCU*, *trnS-GGA*, *trnS-UGA*, *trnT-GGU*, *trnT-UGU*, *trnV-GAC*, trnV-UAC*, *trnW-CCA*, *trnY-GUA*, *trnfM-CAU*
Photosynthesis	Subunits of photosystem I	*psaA*, *psaB*, *psaC*, *psaI*, *psaJ*
	Subunits of photosystem II	*psbA*, *psbB*, *psbC*, *psbD*, *psbE*, *psbF*, *psbH*, *psbI*, *psbJ*, *psbK*, *psbL*, *psbM*, *psbN*, *psbT*, *psbZ*
	Subunits of NADH dehydrogenase	ndhA*, ndhB*, *ndhC*, *ndhD*, *ndhE*, *ndhF*, *ndhG*, *ndhH*, *ndhI*, *ndhJ*, *ndhK*
	Subunits of cytochrome b/f complex	*petA*, petB*, petD*, *petG*, *petL*, *petN*
	Subunits of ATP synthase	*atpA*, *atpB*, *atpE*, atpF*, *atpH*, *atpI*
	Large subunit of rubisco	*rbcL*
Other genes	Maturase	*matK*
	Protease	clpP** (*)
	Envelope membrane protein	*cemA*
	Acetyl-CoA carboxylase	*accD*
	c-type cytochrome synthesis gene	*ccsA*
Unknown	Conserved hypothetical chloroplast ORF	*ycf1*, *ycf2*, ycf3**, *ycf4*

Gene*: Gene with one introns; Gene**: Gene with two introns; Gene (2): Number of copies of multi-copy genes. (*): Gene with one introns in *C. turkestanica*

### Analyses of repeats and simple sequence repeat (SSR)

**Fig. 3:**
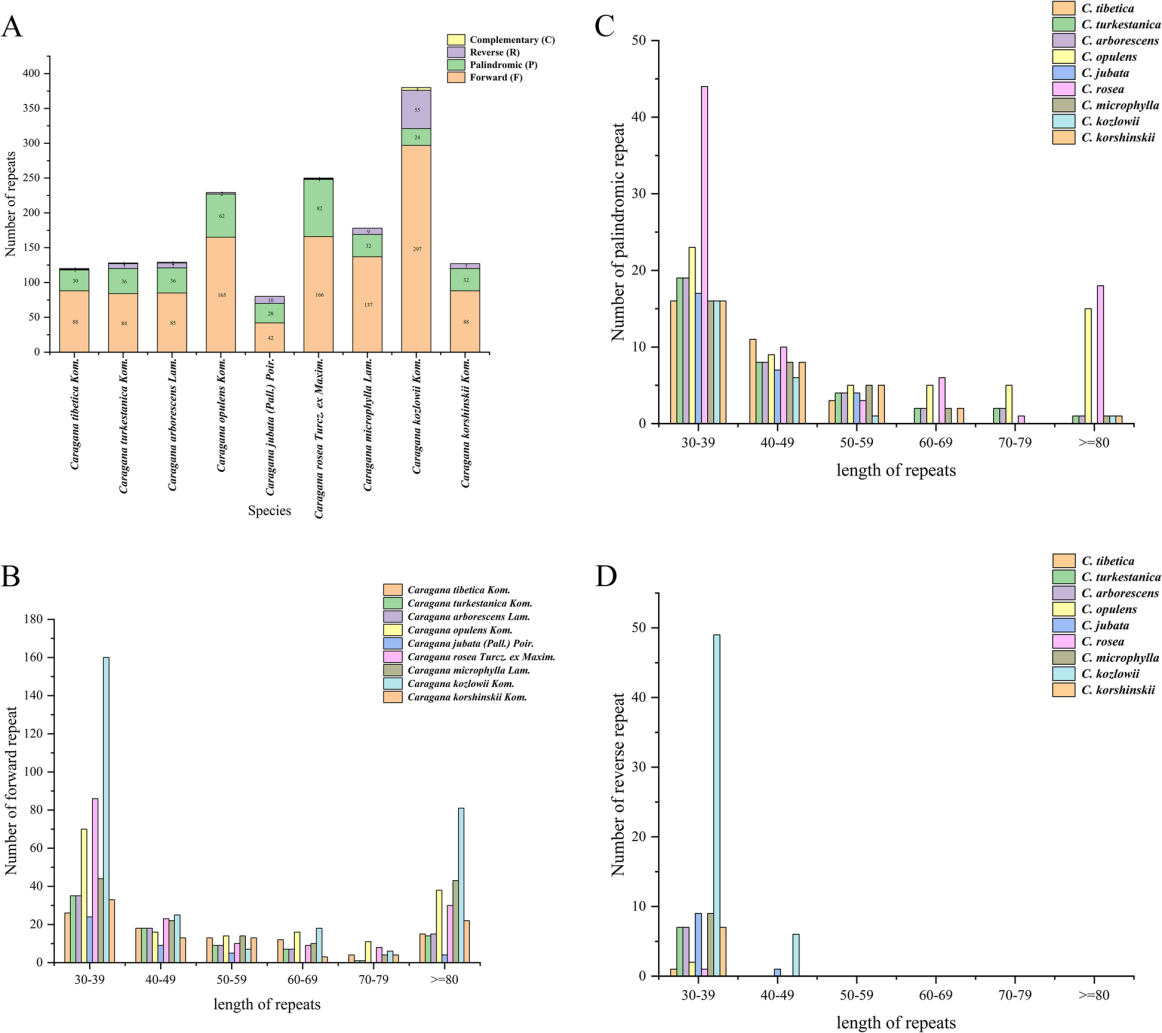
Repeat sequences analysis of 9 *Caragana* cp genomes. **A** The total number of four types of repeat sequences in 9 *Caragana* species; **B** The frequency of forward repeats by length; **C** The frequency of palindrome repeats by length; **D** The frequency of reverse repeats by length

Repetitive units played a critical role in evolution of the genome by facilitating genetic variation and diversity. Through size evolution and structural rearrangements, they promoted genomic mutations and diversity, providing the opportunity for organisms to adapt to new environments and develop new functionalities [38–40]. In the present study, we identified the repetitive sequences present in the cp genomes of *C. tibetica* and *C. turkestanica*, and analyzed content of the two plants. In the genomes of *C. tibetica* and *C. turkestanica*, a total of 119 (length range: 30–337 bp) and 128 (length range: 30–249 bp) repetitive sequences were identified, respectively, consisting of forward (F), palindromic (P), reverse (R), and complementary (C) repeats (Additional file 1: Table S1). In *C. tibetica* and *C. turkestanica*, repetitive analysis detected 88 and 84 forward repeats, 30 and 36 palindromic repeats, 1 and 7 reverse repeats, and 0 and 1 complementary repeats, respectively (Fig. 3A). Among all types of repeats, the frequency of occurrence was highest for sequences with a length ranging from 30 to 49 base pairs (bp). In *C. tibetica*, there were 44 forward repeats, 27 palindromic, and 1 reverse repeats with lengths ranging from 30 to 49 base pairs (Fig. 3B-D). Similarly, in *C. turkestanica*, 53 forward repeats, 27 palindromic, 7 reverse repeats and 1 complementary repeats were 30–49 bp in length (Fig. 3B-D).

In addition, 129, 229, 80, 259, 178, 380, and 127 repeats were found in the reported *C. arborescens*, *C. opulens*, *C. jubata*, *C. rosea*, *C. microphylla*, *C. kozlowii*, and *C. korshinskii* cp genomes, respectively (Additional file 1: Table S1, Fig. 3A). This finding suggested that *C. tibetica* and *C. turkestanica* have a higher degree of similarity in repeat frequency with *C. arborescens* and *C. korshinskii*.

**Fig. 4:**
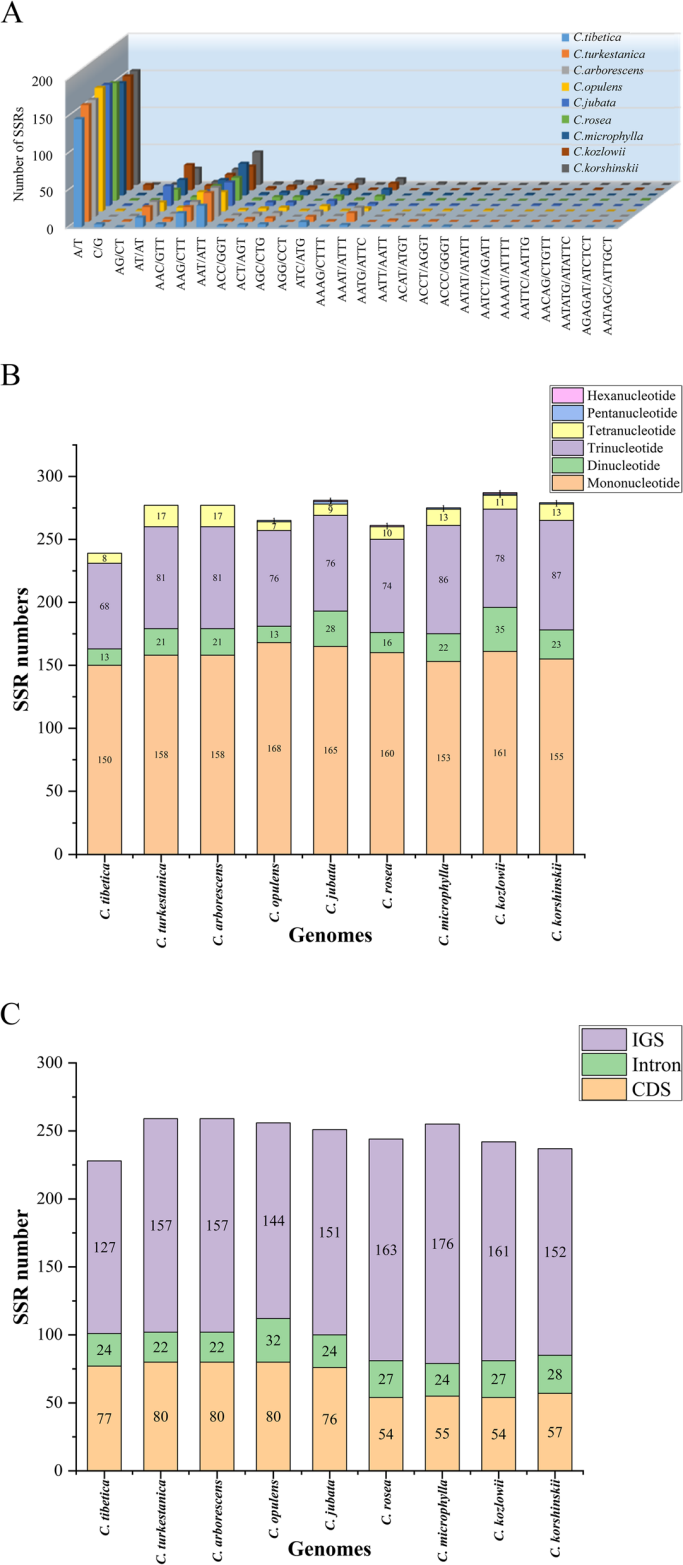
Statistics of SSRs detected in the plastome of nine *Caragana* species. **A** Number of SSRs determined in different repetition types; **B** The amount of different SSR types found in nine *Caragana* species genomes; and **C** The number of SSRs were found in coding (CDS), and intronic regions, intergenic (IGS), Respectively

Simple Sequence Repeat (SSR) loci exhibit extensive and highly variable polymorphism within the genome. As a result, SSRs are considered as effective molecular markers for investigating genetic variations and individual genetic relationships within the genome [41–43]. In this study, we identified intact SSRs in the chloroplast genomes of *C. tibetica* and *C. turkestanica* together with seven additional *Caragana* species (Fig. 4A-C). Based on the propensity of SSRs with 10 bp or longer to undergo slippage and mismatch in the DNA chain, specific parameters have been set to address this phenomenon, which is considered the primary mechanism for SSR polymorphism [43]. We detected a total of 27 types in the two *Caragana* plants, using software MISA (Fig. 4A). Among these, 239 and 277 SSRs loci were detected in *C. tibetica* and *C. turkestanica*. Similarly, we found 277 SSRs in *C. arborescens*, 265 SSRs in *C. opulens*, 281 SSRs in *C. jubata*, 261 SSRs in *C. rosea*, 275 SSRs in *C. microphylla*, 287 SSRs in *C. kozlowii*, and 279 SSRs in *C. korshinskii* (Additional file 1: Table S2). The SSRs in these chloroplast genomes were mainly composed of mononucleotide and trinucleotide repeats motifs. The mononucleotide repeats (A/T and C/G) varied from 150 (62.76%) in *C. tibetica* to 168 (63.64%) in *C. opulens*, while varying from 68 (28.45%) in *C. tibetica* to 86 (31.16%) in *C. microphylla* for trinucleotide repeats (AT/AT and AG/CT) (Fig. 4B, Additional file 1: Table S2). Among them, A/T repeat sequences were the most abundant SSRs. There were 146 and 158 SSRs containing A or T, respectively, in the sequenced species, while only 1 contains C or G.

In addition, there were two pentanucleotide repeats in *C. jubata*, with one present in *C. opulens*, *C. microphylla*, *C. kozlowii* and *C. korshinskii*, one hexanucleotide repeats was found in *C. jubata*, *C. rosea* and *C. kozlowii* using our identification criteria (Fig. 4B, Additional file 1: Table S2). Furthermore, we analyzed the distribution of SSRs in the coding and non-coding regions. The results showed that the number of SSRs in protein-coding regions was significantly lower compared to the non-coding regions (Fig. 4C). We have discovered that the *clpP* gene in *C. tibetica* contains the longest simple repeat sequence, which was a mononucleotide repeat sequence with a length of 49 bp, whereas, the longest SSR was found on the *ycf1* gene in *C. turkestanica*, and it was a single nucleotide repeat sequence with a length of 46 bp ( Additional file 1: Table S3).

### Codon usage bias analysis

During the process of biological evolution, there is a widespread codon usage bias observed in plastids. By analyzing codon usage bias, it is possible to uncover phylogenetic relationships between organisms and molecular evolution of genes, thereby providing potential insights into the origins, mutation patterns, and evolution of species [44, 45]. We have compiled the codon usage information for the protein-coding sequences of nine species (Additional file 1: Table S4). *C. tibetica* and *C. turkestanica* presented the 63 RSCU, and composed of 21,965 and 22,035 codons. There were 21,998 (*C. arborescens*), 22,035 (*C. opulens*), 21,998 (*C. jubata*), 41,710 (*C. rosea*), 40,657 (*C. microphylla*), 41,030 (*C. kozlowii*) and 40,552 (*C. korshinskii*) codons, respectively. Leucine was the amino acid with the highest quantity (2,333 codons in *C. tibetica*, 2,347 codons in *C. turkestanica*, 2,327 codons in *C. arborescens*, 2,347 codons in *C. opulens*, 2,331 codons in *C. jubata*, 4,584 codons in *C. rosea*, 4,195 codons in *C. microphylla*, 4,270 codons in *C. kozlowii* and 4,116 codons in *C. korshinskii*), while the least prevalent were cysteine (259 codons in *C. tibetica*, 261 codons in *C. turkestanica*, 257 codons in *C. arborescens*, 261 codons in *C. opulens*, 258 codons in *C. jubata*) and Tryptophan (656 codons in *C. rosea*, 670 codons in *C. microphylla*, 630 codons in *C. kozlowii* and 639 codons in *C. korshinskii*.

Meanwhile, we also calculated the Relative Synonymous Codon Usage (RSCU) values to assess the codon usage preference in nine *Caragana* species. Using a threshold of 1, codons with RSCU values over 1 were considered as optimal codons. 30 preferred and 32 non-preferred (RSCU < 1.00) codon usages were detected in five species, which were *C. tibetica* (Additional file 2: Fig. S1), *C. turkestanica* (Additional file 2: Fig. S2), *C. arborescens* (Additional file 2: Fig. S3), *C. opulens* (Additional file 2: Fig. S4), and *C. jubata* (Additional file 2: Fig. S5), 28 preferred and 33 non-preferred in *C. rosea* (Additional file 2: Fig. S6) and *C. korshinskii* (Additional file 2: Fig. S7), 29 preferred and 32 non-preferred in *C. microphylla* (Additional file 2: Fig. S8), 31 preferred and 30 non-preferred in *C. kozlowii* (Additional file 1: Table S4, Additional file 2: Fig. S9). Furthermore, the RSCU values for most A/U-ending codons were > 1, while C/G-ending codons were < 1 (Additional file 1: Table S2).

### Sequence divergence analysis

**Fig. 5:**
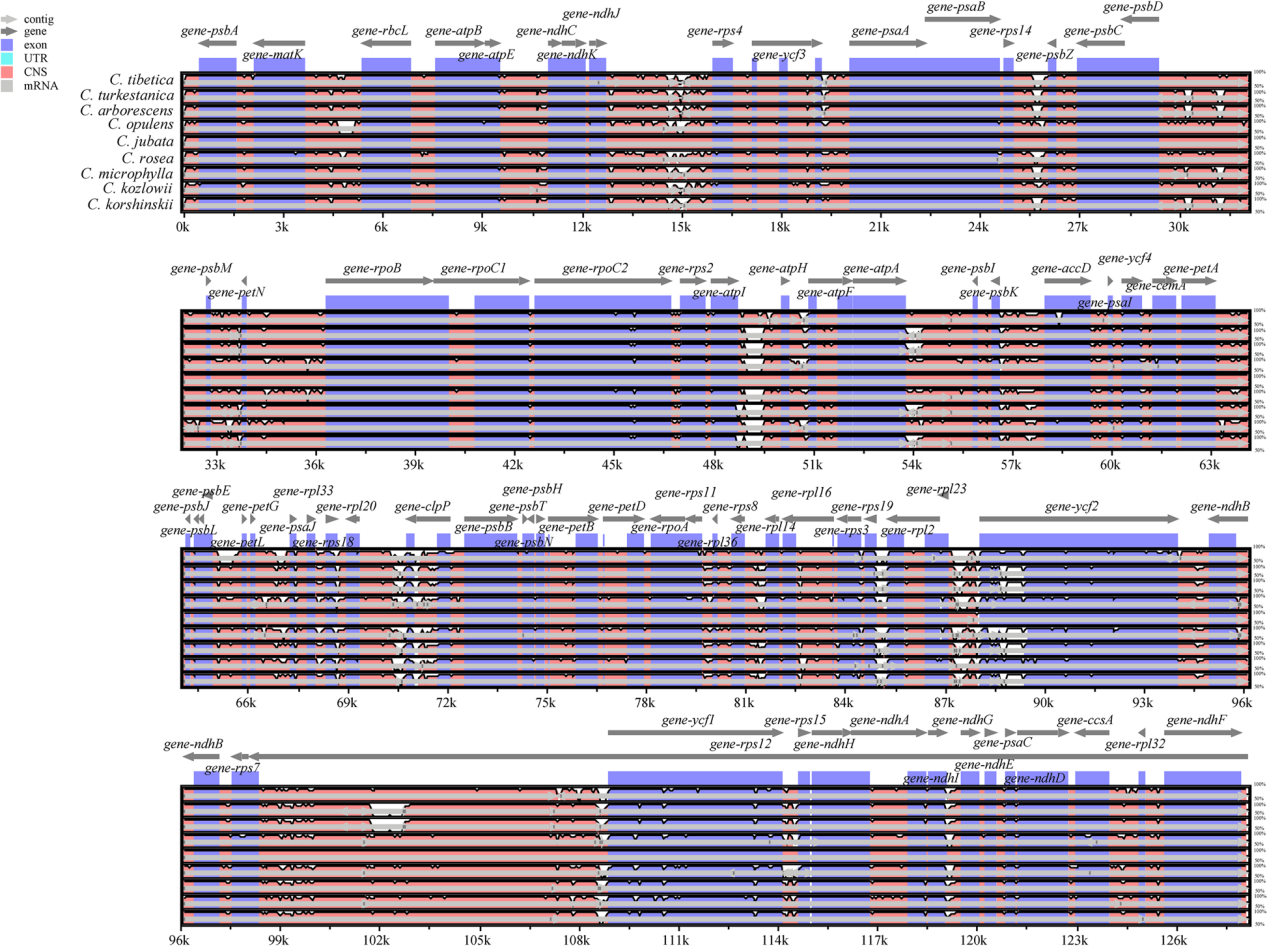
The chloroplast genome of nine *Caragana* species were compared by mVISTA. The gray arrow in the figure indicates the direction of gene translation; The x-axis represents the coordinates in the chloroplast genome; The y-axis represents the percentage between 50 and 100%; Blue indicates protein coding (exon); Light green indicates untranslated region (UTR); Orange indicates conserved non-coding sequences (CNSs)

To reveal the conservative character and divergence in *Caragana* species, we used mVISTA to compare the plastid sequences of *C. tibetica* and *C. turkestanica* studied in this paper and other seven species of *Caragana* plants that have been reported. The annotated chloroplast genome sequence of *C. jubata* served as the reference sequence (Fig. 5). The results revealed a high degree of similarity in the nine plastid genome sequences. However, the sequences were found to exhibit differences in the intergenic spacer (IGS) regions of certain genes, such as *matK-rbcL*, *psbD-psbM*, *atpA-psbI*, and etc. In addition, most of the protein-coding gene sequences were highly conserved, except for a few genes (*rpoC2*, *accD*, *ycf2*, *and ycf1*). Furthermore, compared to the non-coding regions, the coding regions were more conserved. This suggested that the rapidly evolving regions in *Caragana* genus were located in IGS.

**Fig. 6:**
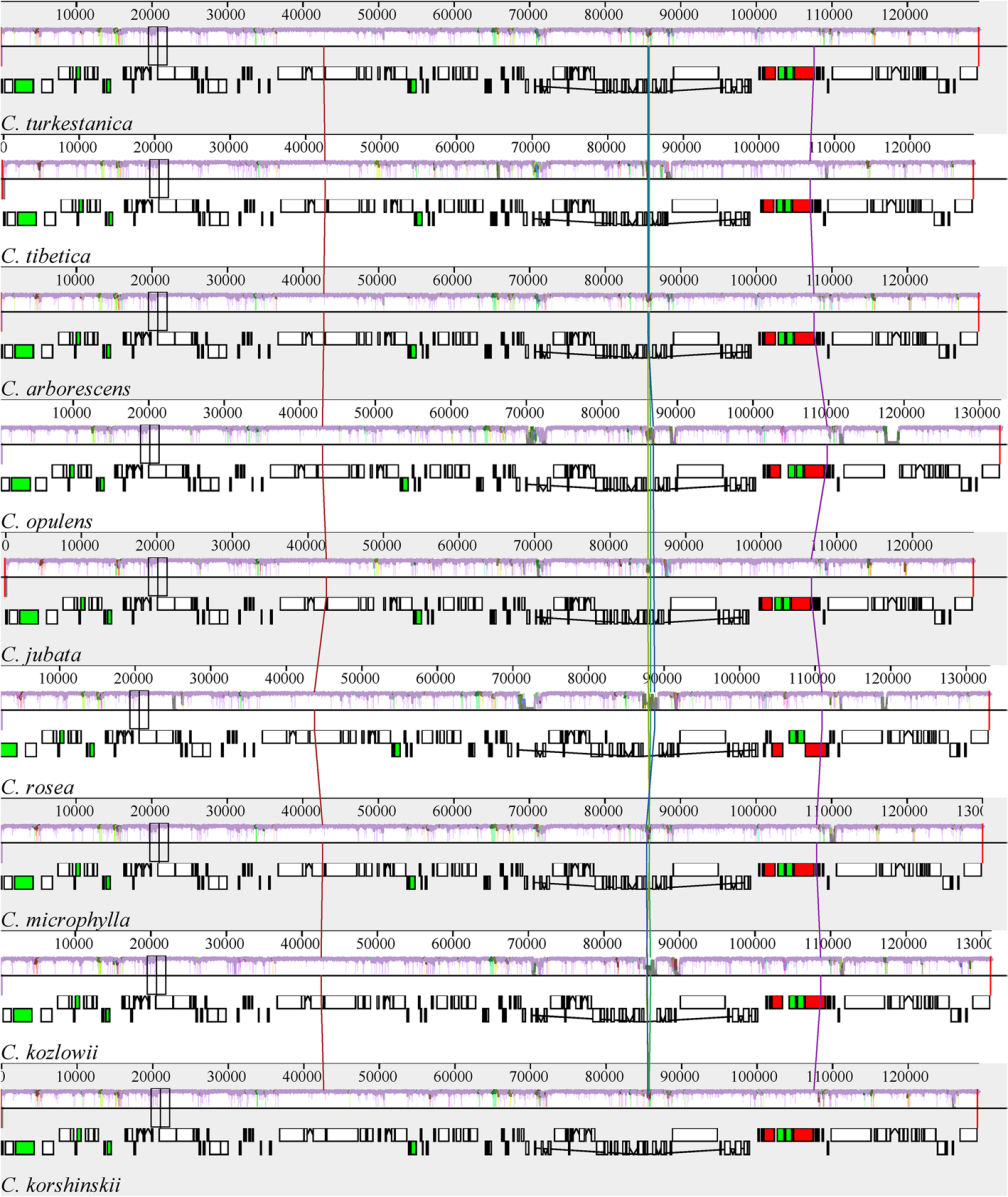
Comparative genomic analysis of chloroplast genome of nine *Caragana* species. Note: Color bands represent genes, and different colors represent different blocks. The squares with the same color between different genes represent homologous regions. In each block, the similarity map of the genome sequence was drawn by Mauve software, and the height of the similarity map corresponded to the average conservative level of the genome sequence region. The two rows below the color band represent the gene. The upper side is on the positive chain, and the lower side is on the complementary chain. The white squares represent CDS, the thin lines in the white squares represent introns, and the green and red squares represent tRNA and rRNA

We utilized the Mauve software to analyze the chloroplast DNA rearrangements in nine species of the genus *Caragana*. The alignment results revealed a high degree of consistency in the types, numbers, and arrangements of coding genes, including CDS, tRNA, and rRNA, across these plants, with no structural inversions or gene rearrangement events observed (Fig. 6).

**Fig. 7:**
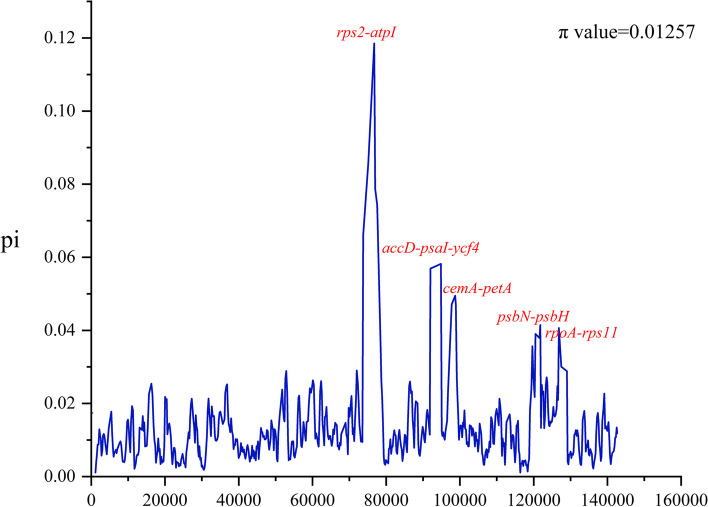
Nucleotide variability (π) values of nine *Caragana* plants. The linear gene graph spectrum of *Caragana* species is given below

Then, we utilized DNAsp6 to detect nucleotide diversity, and identified highly mutated regions in the chloroplast genomes of nine *Caragana* species. The pi values range from 0 to 0.11847, with an average of approximately 0.01257 (Fig. 7), indicating significant differences among the sequences. We have identified five regions that were most likely to be variable, including *rps2-atpI* (π = 0.11847), *accD-psaI-ycf4* (π = 0.05819), *cemA-petA* (π = 0.04949), *psbN-psbH* (π = 0.04144) and *rpoA-rps11* (π = 0.04065). Among these regions, the *rps2-atpI* region had the highest π value (0.11847).

### Phylogenetic analysis

To investigate the phylogenetic positions of 9 species of genus *Caragana* in the family Fabaceae, we conducted phylogenetic analysis using Maximum Likelihood (ML) and Bayesian Inference (BI) methods. Except for the genus *Caragana*, the remaining 8 genus included *Wisteria* (1), *Glycyrrhiza* (2), *Astragalus* (1), *Calophaca* (1), *Cicer* (1), *Medicago* (3), *Trifolium* (3), and *Lathyrus* (4). The number in parentheses represents the number of species in the corresponding taxa.

**Fig. 8:**
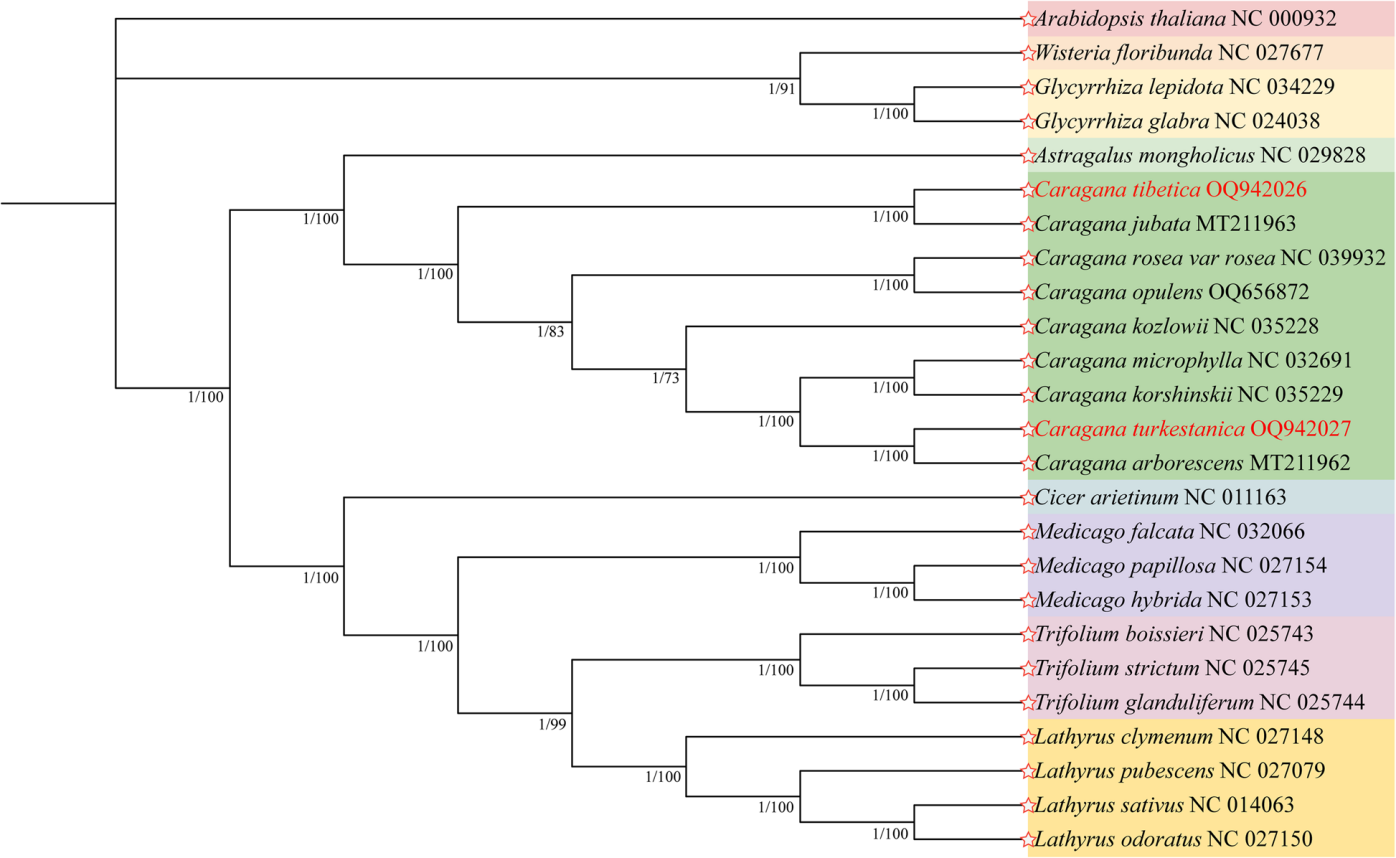
A phylogenetic tree based on the chloroplast genomes of 24 Fabaceae family and one outgroup *Arabidopsis thaliana* was constructed using BI and ML methods. The numbers following the nodes represent bootstrap values. GenBank accession numbers were provided after each species. *C. tibetica* and *C. turkestanica* were highlighted in red

The phylogenetic trees obtained from two methods showed similar topology, and the different datasets generally yielded consistent phylogenetic trees with strong support values. The phylogenetic analysis revealed that all samples were divided into three major branches. Four pairs of species showed closer relationships: *C. tibetica* and *C. jubata*, *C. rosea* and *C. opulens*, *C. microphylla* and *C. korshinskii*, and *C. turkestanica* and *C. arborescens* (Fig. 8). Notably, the close relationship between the genus *Astragalus* and genus *Caragana* (bootstrap: 100%) belonging to the *Subtribe Astragaliinae* was worth mentioning.

## Discussion

This study presented the assembly and annotation of two complete chloroplast genomes. By analyzing these genomes, we gained in-depth insights into the chloroplast genomes of the *Caragana* genus and conducted comparative studies on seven previously reported *Caragana* species.

Reports indicate that researcher, by analyzing characteristics such as morphological features, chromosomes, and pollen morphology of 72 species within the *Caragana* genus and employing cladistic methods, successfully classified these species into 12 series and 5 groups [46]. However, this classification method based on morphology could be influenced by environmental changes and convergent evolution, leading to potential discrepancies in the classification outcomes. With the advancement of molecular marker technology, researcher further utilized DNA sequences from the *rbcL* gene, *trnS-trnG* introns and spacer regions, and the ITS region to study the phylogenetic relationships between 12 *Caragana* species and 48 other legume plants [47]. Although molecular markers have improved the accuracy of classifications in certain aspects, their limitations still exist, preventing the resolution of some evolutionary disputes. Given the chloroplast genome’s low mutation rate, ease of sequencing, and high sequence conservation, it has become a crucial tool for analyzing genetic differences among closely related species, helping to overcome the limitations of traditional methods and enhance classification accuracy [48].

In contrast to the majority of angiosperms, both *C. tibetica* and *C. turkestanica*, along with previously reported *Caragana* species, showed a notable absence of the inverted repeat (IR) region. This absence leaded to an unclear demarcation between the large single-copy (LSC) and small single-copy (SSC) regions. For example, *C. rosea*, *C. microphylla*, *C. intermedia*, *C. jubata*, *C. erinacea*, *C. opulens*, and *C. bicolor* [1, 16, 17, 19]. Comparative analysis of chloroplast genomes within the *Caragana* genus was conducted. The lengths of the chloroplast genomes varied to some degree among the nine *Caragana* plants, with the longest being 133,122 bp in *C. rosea* and the shortest being only 128,132 bp in *C. jubata*. *C. tibetica* and *C. turkestanica* encoded 110 and 111 genes respectively, including 76/76 protein-coding genes, 4/4 rRNA genes, and 30/31 tRNA genes. In this study, we identified a unique gene, *trnN-GUU*, in the genome of *C. turkestanica*, which was missing in the genome of *C. tibetica*. The *trnN-GUU* gene encoded a tRNA that transports valine, representing a unique sequence within *C. turkestanica*’s genome. Through codon usage bias analysis, we discovered that the RSCU of valine-related codons encoded by trnN-GUU in *C. turkestanica* genome exceeds 1, indicating that the use of valine in relation to trnN-GUU may be elevated during protein synthesis. In contrast, no homologous sequence for the *trnN-GUU* gene was identified in *C. tibetica* genome, suggesting that the two subspecies may have different evolutionary histories and paths of adaptive evolution. The presence or absence of *trnN-GUU* in the genomes of *C. turkestanica* and *C. tibetica*, respectively, may reflect their divergent evolutionary branches. Future research should investigate the functional role of the *trnN-GUU* gene in the adaptive evolution of *C. turkestanica*, as well as its evolutionary importance in adaptation to various environments. Additionally, 239 and 277 SSRs were found to be randomly distributed in their genomes. Furthermore, approximately 88/84 forward repeats, 30/36 palindromic repeats, 1/7 reverse repeats, and 0/1 complementary repeats were identified in both cp genomes. We have identified 17 genes containing introns in both chloroplast genomes, with 15 genes containing 1 intron each in *C. tibetica* (16 in *C. turkestanica*), while the genes *clpP* and *ycf3* contained 2 introns in *C. tibetica*, *C. turkestanica* only had one gene *ycf3* contain 2 introns. Introns play a significant role in gene expression regulation, and recent studies have indicated that different introns can enhance the expression of exogenous genes at specific locations [49–51]. In transgenic mice, the addition of an intron from the rabbit-globin gene was found to enhance the expression of the human growth hormone gene, leading to increased levels of the hormone [52]. Similarly, research indicates that deleting the introns of the Drosophila alcohol dehydrogenase (*Adh*) gene leads to reduced expression levels of the *Adh* gene, whether measured by enzymatic activity or RNA levels. This accentuates the important role of introns in the regulation of gene expression [53]. It was found that *ycf2* [54], *rpl23*, and *accD* were frequently missing in some plants, but they were detected in chloroplast genome of *Caragana* plants reported in this paper [55–57]. Previous research has indicated that specific *Caragana* species’ chloroplast genome, including *C. kozlowii*, *C. korshinskii*, *C. microphylla*, and *C. rosea*, exhibit a loss of genes such as *rps16*, *infA*, *rpl22*, and *ycf15* [1]. Similarly, this study also did not find the *rps16*, *infA*, *rpl22*, and *ycf15* loci in *C. tibetica* and *C. turkestanica*. Among these genes, *infA* is an unusually unstable flowering plant chloroplast gene, whereas *rpl22* encodes the ribosomal protein *CL22* and has been eliminated from the chloroplast genome, relocating to the nucleus [58, 59]. Nonetheless, recent studies have discovered that the *infA* gene is present in *C. jubata*, *C. erinacea*, *C. opulens*, and *C. bicolor* [4]. These findings strongly indicated that the *infA* gene might not be lost in some *Caragana* species. Due to the scarcity of available chloroplast genomic data for *Caragana*, it is imperative to conduct extensive experimental research in order to validate these conclusions.

A total of 119 and 128 repeats were detected in the chloroplast genomes of *C. tibetica* and *C. turkestanica*, respectively. These repeats include forward, palindromic, reverse, and complementary sequences. These repeats serve as crucial genetic markers and are closely associated with species emergence and development [60]. Repeat sequences are highly valuable in phylogenetic investigations and also contribute to genome rearrangements [61, 62]. Furthermore, the analysis of various cp genomes has established the vital role of repeat sequences in indel and substitution events [63]. Moreover, no rearrangements have been observed in the plastids of *C. tibetica* and *C. turkestanica*. Previous studies have reported the absence of the IR region in several *Caragana* species, such as *C. microphylla*, *C. bicolor*, and *C. jubata* [1, 16]. Similarly, the cp genomes of *C. tibetica* and *C. turkestanica* were found to lack the IR region in this study. Additionally, the G/C content of the chloroplast DNA (cpDNA) is crucial for determining inter-specific affinity [16]. The DNA G/C content of the two *Caragana* species discussed in this paper is highly alike. Additionally, SSRs are regarded as vital molecular markers for analyzing genetic variation within populations, and they are widely employed in assessing phylogenetic relationships, evolution, and genetic diversity [64]. A total of 239 to 277 SSRs were found in the chloroplast genomes of two *Caragana* plants, exhibiting a significant bias towards A/T. The majority of SSR types consist of single nucleotide repeats, and the highest number of SSRs was observed in the non-coding regions (IGSs) of the cp genomes. These SSRs are valuable starting points for the development of genetic markers in *Caragana* species, and their utilization is applicable in phylogenetic and ecological research.

Codon usage preferences are known to reflect the species of origin and the mutational model. Analyzing patterns of codon usage bias in chloroplast genomes can provide insights into plant phylogenetic relationships, gene expression mechanisms, and molecular evolution [44]. Leucine (Leu) is the most abundant amino acid in *C. tibetica*, *C. turkestanica*, and other *Caragana* species. Furthermore, our research found that the majority of synonymous codons preferred for RSCU values end with A/U, thereby contributing to a higher AT content in genes. Based on our analysis, we hypothesize that natural selection and gene mutation may be responsible for this codon usage pattern. Nevertheless, it is important to note that codon preference and utilization patterns provide only a partial reflection of the evolutionary relationship between species, and further research is necessary.

The plastid genome contains numerous variable nucleotides, which can be utilized as valuable DNA barcodes for determining the relationships between species or genera [65, 66]. We simultaneously identified five intergenic spacer regions (IGSs) with relatively high differentiation values: *rps2-atpI*, *accD-psaI-ycf4*, *cemA-petA*, *psbN-psbH*, and *rpoA-rps11*. These variable regions have the potential to function as DNA barcodes for studying phylogenetic relationships, species identification, and population genetics research. Next, we compared the sequence variations of the nine assembled *Caragana* plants. Comparative analysis of the chloroplast genomes confirmed that the coding regions were more conserved than the non-coding regions, which is consistent with findings from other *Caragana* species [16].

Recently, the chloroplast genome has become a preferred option for studying the phylogenetic relationships of diverse plant species. For instance, the utilization of the complete chloroplast genomes from three *Lycoris* plants in phylogenetic analysis revealed a strongly supported relationship between *Lycoris* plants and the *Narcissus* genus [67]. Phylogenetic analysis of 23 *Swertia* plant species revealed that *Swertia* is paraphyletic rather than monophyletic [68]. The phylogenetic position of *C. tibetica* and *C. turkestanica* within Fabaceae was determined by constructing a comprehensive genomic dataset composed of 66 genes shared among 24 representatives from nine genera. The phylogenetic analysis indicated that *Caragana* species constituted a distinct clade, with *C. microphylla* and *C. korshinskii* showing a closer genetic relationship, which aligns with earlier findings [1, 16]. The phylogenetic relationships inferred from the chloroplast genome offer novel insights and perspectives to advance our understanding of plant evolution.

For the first time, we assembled and analyzed the complete chloroplast genomes of *C. tibetica* and *C. turkestanica*, and compared them to other members of the *Caragana* genus. Our findings revealed that the sizes of their genomes, gene compositions, gene arrangements, GC content, and codon usage were similar to previously documented chloroplast genomes within the *Caragana* genus. Additionally, this study identified the positions and distributions of repetitive sequences in both species, and determined the sequence variability and nucleotide variation sites. The results of this study provide guidance for future studies on the phylogenetic evolution and species identification of the *Caragana* genus, as well as the development of new molecular markers. Ultimately, this discovery contributes to the augmentation of the chloroplast genome database for the *Caragana* genus.

## Conclusions

Our study presents the initial assembly and annotation of *C. tibetica* and *C. turkestanica* and compares them with seven other *Caragana* species. Due to the absence of a pair of IRs, the plastomes of *C. tibetica* and *C. turkestanica* were shorter, with sizes ranging from 128,433 bp to 129,453 bp. The long repeats, SSR loci, and certain genes found in the IGS region (*matK-rbcL*, *psbD-psbM*, *atpA-psbI*) as well as five regions exhibiting high variability (*rps2-atpI, accD-psaI-ycf4, cemA-petA, psbN-psbH*, and *rpoA-rps11*) identified in our study will promote future research efforts. These efforts include the development of new molecular markers and investigations of population genetics and phylogenetic analysis. By analyzing the sequence and structural information of the chloroplast genomes of two *Caragana* plants comprehensively, we have determined their genetic evolutionary position and relationships with other *Caragana* species. This information establishes a foundation for extensive and detailed research on the identification, genetic diversity, and phylogenetics of *Caragana* species. Furthermore, our study has significantly contributed to the enrichment of the chloroplast genome database for *Caragana* plants.

## Methods

### Plant material, DNA extraction and sequencing

The fresh and young leaves of *C. tibetica*, and *C. turkestanica* were gathered from eastern part of Qinghai Province (N36°43′24.80′′, E101°44′54.11′′), China. During outdoor sampling, the leaf tissues were temporarily stored in a low-temperature insulated box. After returning to the laboratory, the samples were immediately placed in a -80℃ ultra-low temperature freezer for storage. We used a modified cetyltrimethylammonium bromide (CTAB) [69] method to extract DNA from fresh tissue samples of *Caragana* plants. Ultrasound was used to fragment the DNA fragments, and the fragment size was selected by agarose gel electrophoresis. The selected fragments were amplified by PCR to form a sequencing library, and the qualified library was sequenced using the Illumina NovaSeq platform, with 150 bp pair-end reads.

### Gene annotation, genome assembly and sequence analyses

Before assembly, we conducted a rigorous preprocessing of the raw data. The raw data were filtered using the Trimmomatic v 0.39 [70] tool to remove low-quality data. After that, we used SPAdes v3.10.1 (http://cab.spbu.ru/software/spades/) [71] to assemble chloroplast genome sequences to obtain their SEED sequences, K-mer analysis was conducted on the seed sequence to obtain Contigs. We employed SSPACE v2 [72] for the assembly of contig sequences into scaffolds. Subsequently, Gapfiller v2.1.1 [73] was utilized to resolve any gaps within these scaffolds, thereby enhancing the coherence and completeness of the pseudo-genome assembly. Then, based on the structure of the chloroplast genome, the corrected pseudo genome sequences were reordered and aligned, resulting in two complete circular chloroplast genome sequences. After assembly, quality control of the final sequence was performed using the reference sequence for *C. kozlowii* in this study. The annotation information for the CDS, rRNA, and tRNA sequences in the chloroplast genome were gained using Blast v2.2.25 (https://blast.ncbi.nlm.nih.gov/Blast.cgi), hmmer v3.1b2 [74] (http://www.hmmer.org/), and ARAGORN v1.2.38 [75] (http://130.235.244.92/ARAGORN/) software, respectively. The chloroplast genome maps for *C. tibetica* and *C. turkestanica* were plotted by the online tool Chloroplot in OGDRAW [76]. Finally, the annotated chloroplast genome sequences of the genus *Caragana* were submitted to GenBank using the online submission tool BankIt, and the accession numbers OQ942026 and OQ942027 were obtained, respectively.

### Repeats, simple sequence repeats and codon usage bias analysis

Vmatch [77] was chosen to search for forward, reverse, palindromic, and complementary repeats in the chloroplast genome sequences of *Caragana* species. Additionally, simple sequence repeats (SSRs) in this study were identified by MISA [78] with the following parameter settings: mono-nucleotide set as 8, di-nucleotide set as 5, tri-nucleotide set as 3, tetra-nucleotide set as 3, penta-nucleotide set as 3, and hexa-nucleotide set as 3, respectively. The program CodonW1.4.2 [79] was applied to calculate relative synonymous codon usage (RSCU) values of protein-coding genes under default settings.

### Sequence divergence and comparative genome analysis

The chloroplast genome sequences of *Caragana* species were aligned using MEGA7 [80], and DnaSP6 [81] was used to calculate nucleotide diversity (π) values with the following parameter settings: window length of 600 bp and step size of 200 bp, which are commonly used in the literature. Comparison and visualization of complete *Caragana* chloroplast genomes using mVISTA [82] program (Shuffle-LAGAN mode). *C. jubata* plastome was labeled as reference. Utilizing Mauve software [83], an analysis was conducted on the chloroplast DNA rearrangements of nine species within the genus *Caragana*, aiming to identify changes in gene order, potential large-scale sequence rearrangements, and local tandem duplication events. This step employed the software’s recommended default parameters.

### Phylogenetic analysis

To determine the phylogenetic positions of *Caragana* species in this study, we downloaded plastid genome sequences of 24 legume species which belong to the IRLC, and one outgroup (*Arabidopsis thaliana*) from NCBI. Phylogenetic trees were constructed using PhyloSuite v1.2.2 [84]. First, we extracted 66 common protein-coding gene sequences from each of 25 chloroplast genomes. These protein gene sequences were then aligned in batches using the MAFFT [85] with auto strategy and normal alignment mode. This study constructed phylogenetic trees based on the chloroplast genome using two methods: Bayesian inference (BI) and maximum likelihood (ML). In the Bayesian Inference (BI) analysis, we employed Modelfinder [86] to determine the most suitable model, opting for the GTR + F + I + G4 nucleotide substitution model. Subsequently, phylogenetic trees were inferred using Bayesian inference [87], leveraging a partition model to enhance the accuracy of our findings. It runs in parallel with one execution, performing a total of 1,000,000 generations. During the analysis, the initial 25% of sampled data is discarded as a burn-in period. To ensure the convergence of the Markov Chain Monte Carlo (MCMC) algorithm, these samples are excluded from the final analysis. Additionally, the average standard deviation of split frequencies is set to a threshold greater than 0.01 [88]. In an evolutionary tree constructed using BI, a value of 1 represents the highest probability or maximum posterior probability for support, indicating that a particular structure or branch is highly reliable given the data and model. For ML, the IQ-TREE [89] tool was used, with automatic partition selection and 1,000 ultrafast bootstrap [90]replicates performed to assess the confidence of each branch. And we set the threshold for Bootstrap values at 70% as the criterion for evaluating support. Therefore, when the Bootstrap value of a particular clade reaches or exceeds 70%, it is considered to have strong support.

### Supplementary Information

**Additional file 1: Table S1.** Types and numbers of Repeats in chloroplast genome of 9 *Caragana* spices. **Table S2.** Types and numbers of SSR in chloroplast genome of 9 *Caragana* spices. **Table S3.** Distribution of SSRs in cp genome of *C. tibetica* and *C.turkestanica*. **Table S4.** Analysis of coding ability and codon preference of chloroplast genome of *C. tibetica* and *C.turkestanica*.

**Additional file 2: Fig. S1.** Amino acid frequencies of the chloroplast genomes of *C. tibetica. *The squares below represent all the codons that encode each type of amino acid; the height of the column above represents the total sum of RSCU values for all codons; the height of each column represents the RSCU value for each codon. **Fig. S2.** Amino acid frequencies of the chloroplast genomes of *C. turkestanica. *The squares below represent all the codons that encode each type of amino acid; the height of the column above represents the total sum of RSCU values for all codons; the height of each column represents the RSCU value for each codon. **Fig. S3.** Amino acid frequencies of the chloroplast genomes of *C. arborescens. *The squares below represent all the codons that encode each type of amino acid; the height of the column above represents the total sum of RSCU values for all codons; the height of each column represents the RSCU value for each codon. **Fig. S4.** Amino acid frequencies of the chloroplast genomes of *C. opulens. *The squares below represent all the codons that encode each type of amino acid; the height of the column above represents the total sum of RSCU values for all codons; the height of each column represents the RSCU value for each codon. **Fig. S5.** Amino acid frequencies of the chloroplast genomes of *C. jubata. *The squares below represent all the codons that encode each type of amino acid; the height of the column above represents the total sum of RSCU values for all codons; the height of each column represents the RSCU value for each codon. **Fig. S6.** Amino acid frequencies of the chloroplast genomes of *C. rosea. *The squares below represent all the codons that encode each type of amino acid; the height of the column above represents the total sum of RSCU values for all codons; the height of each column represents the RSCU value for each codon. **Fig. S7.** Amino acid frequencies of the chloroplast genomes of *C. microphylla. *The squares below represent all the codons that encode each type of amino acid; the height of the column above represents the total sum of RSCU values for all codons; the height of each column represents the RSCU value for each codon. **Fig. S8.** Amino acid frequencies of the chloroplast genomes of *C. kozlowii. *The squares below represent all the codons that encode each type of amino acid; the height of the column above represents the total sum of RSCU values for all codons; the height of each column represents the RSCU value for each codon. **Fig. S9.** Amino acid frequencies of the chloroplast genomes of *C. korshinskii. *The squares below represent all the codons that encode each type of amino acid; the height of the column above represents the total sum of RSCU values for all codons; the height of each column represents the RSCU value for each codon.

## Data Availability

The original sequencing data have been submitted to the NCBI database and received GenBank accession numbers OQ942026 (*C. tibetica*), OQ942027 (*C. turkestanica*). The data used in this study are already entirely in the public domain (https://www.ncbi.nlm.nih.gov). Voucher specimens of *C. tibetica* and *C. turkestanica* are stored in the herbarium of the College of Eco-Environmental Engineering at Qinghai University. The voucher specimen number for *C. tibetica* is QhST20190080, and number QhST20190081 for *C. turkestanica*.
