# Supplementary figures and images for: Chloroplast genomes of Caragana tibetica and Caragana turkestanica: structures and comparative analysis
Source: BMC Plant Biol. 2024 Apr 9;24:254. doi: 10.1186/s12870-024-04979-9 (PMC11003120; doi:10.1186/s12870-024-04979-9)

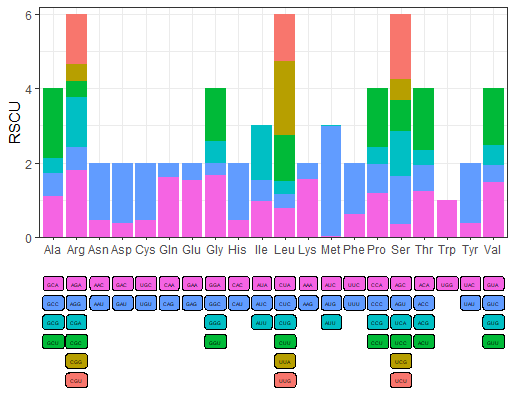

Supplement: Supplementary file 2 — Additional file 2: Fig. S1. Amino acid frequencies of the chloroplast genomes of C. tibetica. The squares below represent all the codons that encode each type of amino acid; the height of the column above represents the total sum of RSCU values for all codons; the height of each column represents the RSCU value for each codon. Fig. S2. Amino acid frequencies of the chloroplast genomes of C. turkestanica. The squares below represent all the codons that encode each type of amino acid; the height of the column above represents the total sum of RSCU values for all codons; the height of each column represents the RSCU value for each codon. Fig. S3. Amino acid frequencies of the chloroplast genomes of C. arborescens. The squares below represent all the codons that encode each type of amino acid; the height of the column above represents the total sum of RSCU values for all codons; the height of each column represents the RSCU value for each codon. Fig. S4. Amino acid frequencies of the chloroplast genomes of C. opulens. The squares below represent all the codons that encode each type of amino acid; the height of the column above represents the total sum of RSCU values for all codons; the height of each column represents the RSCU value for each codon. Fig. S5. Amino acid frequencies of the chloroplast genomes of C. jubata. The squares below represent all the codons that encode each type of amino acid; the height of the column above represents the total sum of RSCU values for all codons; the height of each column represents the RSCU value for each codon. Fig. S6. Amino acid frequencies of the chloroplast genomes of C. rosea. The squares below represent all the codons that encode each type of amino acid; the height of the column above represents the total sum of RSCU values for all codons; the height of each column represents the RSCU value for each codon. Fig. S7. Amino acid frequencies of the chloroplast genomes of C. microphylla. The squares below represent all the codons [file 12870_2024_4979_MOESM2_ESM.zip › Additional file 2/Fig. S1.png]

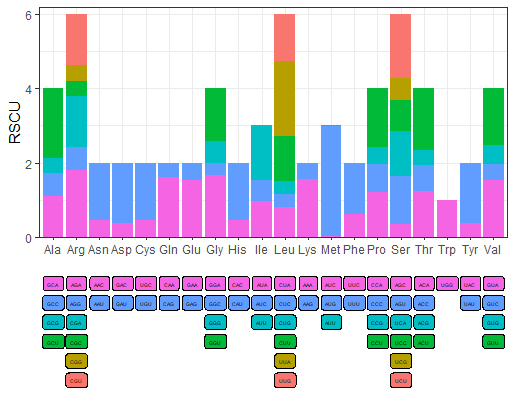

Supplement: Supplementary file 2 — Additional file 2: Fig. S1. Amino acid frequencies of the chloroplast genomes of C. tibetica. The squares below represent all the codons that encode each type of amino acid; the height of the column above represents the total sum of RSCU values for all codons; the height of each column represents the RSCU value for each codon. Fig. S2. Amino acid frequencies of the chloroplast genomes of C. turkestanica. The squares below represent all the codons that encode each type of amino acid; the height of the column above represents the total sum of RSCU values for all codons; the height of each column represents the RSCU value for each codon. Fig. S3. Amino acid frequencies of the chloroplast genomes of C. arborescens. The squares below represent all the codons that encode each type of amino acid; the height of the column above represents the total sum of RSCU values for all codons; the height of each column represents the RSCU value for each codon. Fig. S4. Amino acid frequencies of the chloroplast genomes of C. opulens. The squares below represent all the codons that encode each type of amino acid; the height of the column above represents the total sum of RSCU values for all codons; the height of each column represents the RSCU value for each codon. Fig. S5. Amino acid frequencies of the chloroplast genomes of C. jubata. The squares below represent all the codons that encode each type of amino acid; the height of the column above represents the total sum of RSCU values for all codons; the height of each column represents the RSCU value for each codon. Fig. S6. Amino acid frequencies of the chloroplast genomes of C. rosea. The squares below represent all the codons that encode each type of amino acid; the height of the column above represents the total sum of RSCU values for all codons; the height of each column represents the RSCU value for each codon. Fig. S7. Amino acid frequencies of the chloroplast genomes of C. microphylla. The squares below represent all the codons [file 12870_2024_4979_MOESM2_ESM.zip › Additional file 2/Fig. S2.png]

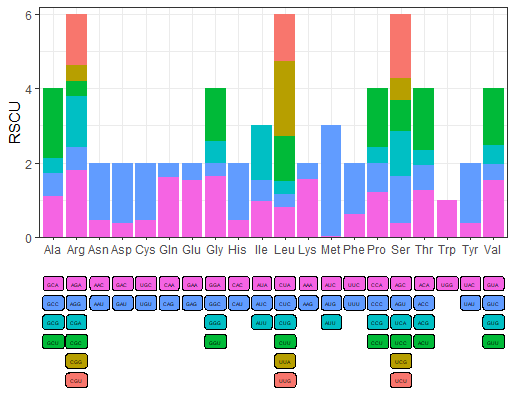

Supplement: Supplementary file 2 — Additional file 2: Fig. S1. Amino acid frequencies of the chloroplast genomes of C. tibetica. The squares below represent all the codons that encode each type of amino acid; the height of the column above represents the total sum of RSCU values for all codons; the height of each column represents the RSCU value for each codon. Fig. S2. Amino acid frequencies of the chloroplast genomes of C. turkestanica. The squares below represent all the codons that encode each type of amino acid; the height of the column above represents the total sum of RSCU values for all codons; the height of each column represents the RSCU value for each codon. Fig. S3. Amino acid frequencies of the chloroplast genomes of C. arborescens. The squares below represent all the codons that encode each type of amino acid; the height of the column above represents the total sum of RSCU values for all codons; the height of each column represents the RSCU value for each codon. Fig. S4. Amino acid frequencies of the chloroplast genomes of C. opulens. The squares below represent all the codons that encode each type of amino acid; the height of the column above represents the total sum of RSCU values for all codons; the height of each column represents the RSCU value for each codon. Fig. S5. Amino acid frequencies of the chloroplast genomes of C. jubata. The squares below represent all the codons that encode each type of amino acid; the height of the column above represents the total sum of RSCU values for all codons; the height of each column represents the RSCU value for each codon. Fig. S6. Amino acid frequencies of the chloroplast genomes of C. rosea. The squares below represent all the codons that encode each type of amino acid; the height of the column above represents the total sum of RSCU values for all codons; the height of each column represents the RSCU value for each codon. Fig. S7. Amino acid frequencies of the chloroplast genomes of C. microphylla. The squares below represent all the codons [file 12870_2024_4979_MOESM2_ESM.zip › Additional file 2/Fig. S3.png]

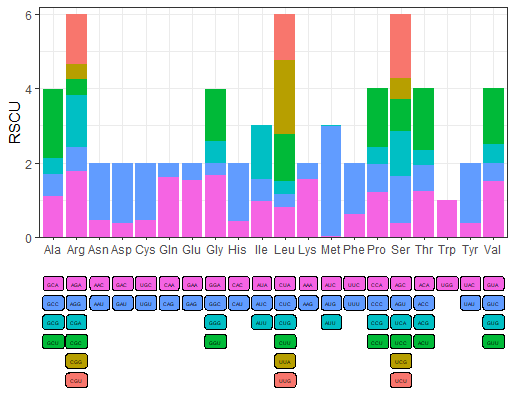

Supplement: Supplementary file 2 — Additional file 2: Fig. S1. Amino acid frequencies of the chloroplast genomes of C. tibetica. The squares below represent all the codons that encode each type of amino acid; the height of the column above represents the total sum of RSCU values for all codons; the height of each column represents the RSCU value for each codon. Fig. S2. Amino acid frequencies of the chloroplast genomes of C. turkestanica. The squares below represent all the codons that encode each type of amino acid; the height of the column above represents the total sum of RSCU values for all codons; the height of each column represents the RSCU value for each codon. Fig. S3. Amino acid frequencies of the chloroplast genomes of C. arborescens. The squares below represent all the codons that encode each type of amino acid; the height of the column above represents the total sum of RSCU values for all codons; the height of each column represents the RSCU value for each codon. Fig. S4. Amino acid frequencies of the chloroplast genomes of C. opulens. The squares below represent all the codons that encode each type of amino acid; the height of the column above represents the total sum of RSCU values for all codons; the height of each column represents the RSCU value for each codon. Fig. S5. Amino acid frequencies of the chloroplast genomes of C. jubata. The squares below represent all the codons that encode each type of amino acid; the height of the column above represents the total sum of RSCU values for all codons; the height of each column represents the RSCU value for each codon. Fig. S6. Amino acid frequencies of the chloroplast genomes of C. rosea. The squares below represent all the codons that encode each type of amino acid; the height of the column above represents the total sum of RSCU values for all codons; the height of each column represents the RSCU value for each codon. Fig. S7. Amino acid frequencies of the chloroplast genomes of C. microphylla. The squares below represent all the codons [file 12870_2024_4979_MOESM2_ESM.zip › Additional file 2/Fig. S4.png]

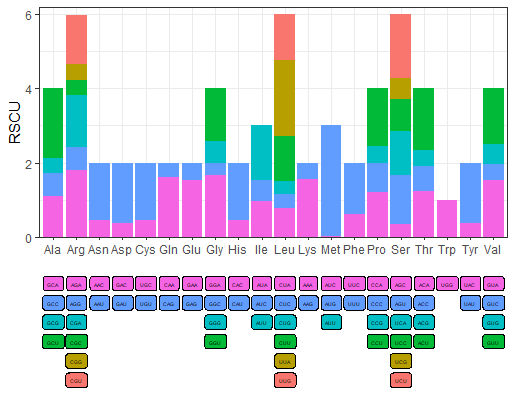

Supplement: Supplementary file 2 — Additional file 2: Fig. S1. Amino acid frequencies of the chloroplast genomes of C. tibetica. The squares below represent all the codons that encode each type of amino acid; the height of the column above represents the total sum of RSCU values for all codons; the height of each column represents the RSCU value for each codon. Fig. S2. Amino acid frequencies of the chloroplast genomes of C. turkestanica. The squares below represent all the codons that encode each type of amino acid; the height of the column above represents the total sum of RSCU values for all codons; the height of each column represents the RSCU value for each codon. Fig. S3. Amino acid frequencies of the chloroplast genomes of C. arborescens. The squares below represent all the codons that encode each type of amino acid; the height of the column above represents the total sum of RSCU values for all codons; the height of each column represents the RSCU value for each codon. Fig. S4. Amino acid frequencies of the chloroplast genomes of C. opulens. The squares below represent all the codons that encode each type of amino acid; the height of the column above represents the total sum of RSCU values for all codons; the height of each column represents the RSCU value for each codon. Fig. S5. Amino acid frequencies of the chloroplast genomes of C. jubata. The squares below represent all the codons that encode each type of amino acid; the height of the column above represents the total sum of RSCU values for all codons; the height of each column represents the RSCU value for each codon. Fig. S6. Amino acid frequencies of the chloroplast genomes of C. rosea. The squares below represent all the codons that encode each type of amino acid; the height of the column above represents the total sum of RSCU values for all codons; the height of each column represents the RSCU value for each codon. Fig. S7. Amino acid frequencies of the chloroplast genomes of C. microphylla. The squares below represent all the codons [file 12870_2024_4979_MOESM2_ESM.zip › Additional file 2/Fig. S5.png]

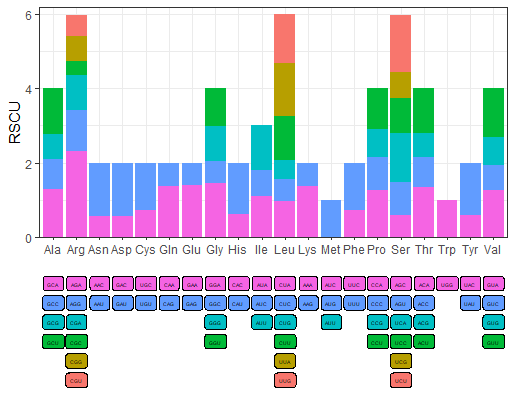

Supplement: Supplementary file 2 — Additional file 2: Fig. S1. Amino acid frequencies of the chloroplast genomes of C. tibetica. The squares below represent all the codons that encode each type of amino acid; the height of the column above represents the total sum of RSCU values for all codons; the height of each column represents the RSCU value for each codon. Fig. S2. Amino acid frequencies of the chloroplast genomes of C. turkestanica. The squares below represent all the codons that encode each type of amino acid; the height of the column above represents the total sum of RSCU values for all codons; the height of each column represents the RSCU value for each codon. Fig. S3. Amino acid frequencies of the chloroplast genomes of C. arborescens. The squares below represent all the codons that encode each type of amino acid; the height of the column above represents the total sum of RSCU values for all codons; the height of each column represents the RSCU value for each codon. Fig. S4. Amino acid frequencies of the chloroplast genomes of C. opulens. The squares below represent all the codons that encode each type of amino acid; the height of the column above represents the total sum of RSCU values for all codons; the height of each column represents the RSCU value for each codon. Fig. S5. Amino acid frequencies of the chloroplast genomes of C. jubata. The squares below represent all the codons that encode each type of amino acid; the height of the column above represents the total sum of RSCU values for all codons; the height of each column represents the RSCU value for each codon. Fig. S6. Amino acid frequencies of the chloroplast genomes of C. rosea. The squares below represent all the codons that encode each type of amino acid; the height of the column above represents the total sum of RSCU values for all codons; the height of each column represents the RSCU value for each codon. Fig. S7. Amino acid frequencies of the chloroplast genomes of C. microphylla. The squares below represent all the codons [file 12870_2024_4979_MOESM2_ESM.zip › Additional file 2/Fig. S6.png]

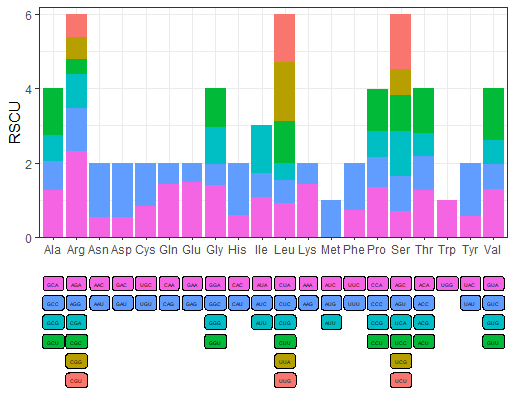

Supplement: Supplementary file 2 — Additional file 2: Fig. S1. Amino acid frequencies of the chloroplast genomes of C. tibetica. The squares below represent all the codons that encode each type of amino acid; the height of the column above represents the total sum of RSCU values for all codons; the height of each column represents the RSCU value for each codon. Fig. S2. Amino acid frequencies of the chloroplast genomes of C. turkestanica. The squares below represent all the codons that encode each type of amino acid; the height of the column above represents the total sum of RSCU values for all codons; the height of each column represents the RSCU value for each codon. Fig. S3. Amino acid frequencies of the chloroplast genomes of C. arborescens. The squares below represent all the codons that encode each type of amino acid; the height of the column above represents the total sum of RSCU values for all codons; the height of each column represents the RSCU value for each codon. Fig. S4. Amino acid frequencies of the chloroplast genomes of C. opulens. The squares below represent all the codons that encode each type of amino acid; the height of the column above represents the total sum of RSCU values for all codons; the height of each column represents the RSCU value for each codon. Fig. S5. Amino acid frequencies of the chloroplast genomes of C. jubata. The squares below represent all the codons that encode each type of amino acid; the height of the column above represents the total sum of RSCU values for all codons; the height of each column represents the RSCU value for each codon. Fig. S6. Amino acid frequencies of the chloroplast genomes of C. rosea. The squares below represent all the codons that encode each type of amino acid; the height of the column above represents the total sum of RSCU values for all codons; the height of each column represents the RSCU value for each codon. Fig. S7. Amino acid frequencies of the chloroplast genomes of C. microphylla. The squares below represent all the codons [file 12870_2024_4979_MOESM2_ESM.zip › Additional file 2/Fig. S7.png]

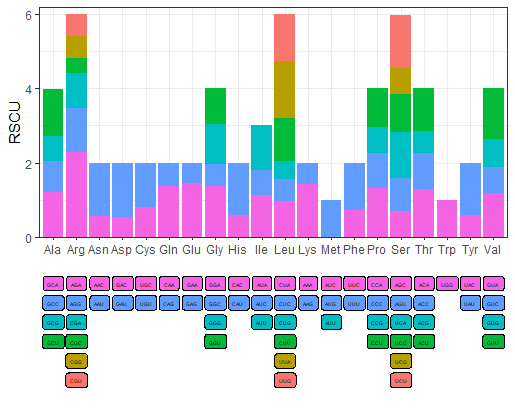

Supplement: Supplementary file 2 — Additional file 2: Fig. S1. Amino acid frequencies of the chloroplast genomes of C. tibetica. The squares below represent all the codons that encode each type of amino acid; the height of the column above represents the total sum of RSCU values for all codons; the height of each column represents the RSCU value for each codon. Fig. S2. Amino acid frequencies of the chloroplast genomes of C. turkestanica. The squares below represent all the codons that encode each type of amino acid; the height of the column above represents the total sum of RSCU values for all codons; the height of each column represents the RSCU value for each codon. Fig. S3. Amino acid frequencies of the chloroplast genomes of C. arborescens. The squares below represent all the codons that encode each type of amino acid; the height of the column above represents the total sum of RSCU values for all codons; the height of each column represents the RSCU value for each codon. Fig. S4. Amino acid frequencies of the chloroplast genomes of C. opulens. The squares below represent all the codons that encode each type of amino acid; the height of the column above represents the total sum of RSCU values for all codons; the height of each column represents the RSCU value for each codon. Fig. S5. Amino acid frequencies of the chloroplast genomes of C. jubata. The squares below represent all the codons that encode each type of amino acid; the height of the column above represents the total sum of RSCU values for all codons; the height of each column represents the RSCU value for each codon. Fig. S6. Amino acid frequencies of the chloroplast genomes of C. rosea. The squares below represent all the codons that encode each type of amino acid; the height of the column above represents the total sum of RSCU values for all codons; the height of each column represents the RSCU value for each codon. Fig. S7. Amino acid frequencies of the chloroplast genomes of C. microphylla. The squares below represent all the codons [file 12870_2024_4979_MOESM2_ESM.zip › Additional file 2/Fig. S8.png]

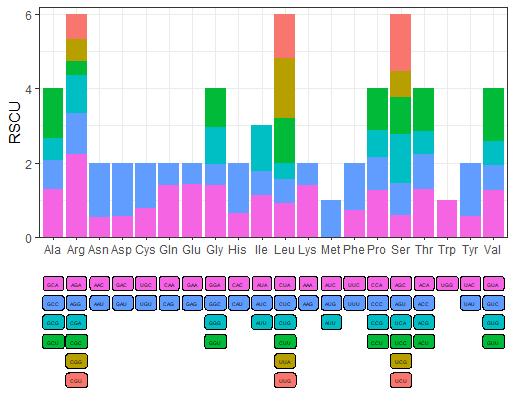

Supplement: Supplementary file 2 — Additional file 2: Fig. S1. Amino acid frequencies of the chloroplast genomes of C. tibetica. The squares below represent all the codons that encode each type of amino acid; the height of the column above represents the total sum of RSCU values for all codons; the height of each column represents the RSCU value for each codon. Fig. S2. Amino acid frequencies of the chloroplast genomes of C. turkestanica. The squares below represent all the codons that encode each type of amino acid; the height of the column above represents the total sum of RSCU values for all codons; the height of each column represents the RSCU value for each codon. Fig. S3. Amino acid frequencies of the chloroplast genomes of C. arborescens. The squares below represent all the codons that encode each type of amino acid; the height of the column above represents the total sum of RSCU values for all codons; the height of each column represents the RSCU value for each codon. Fig. S4. Amino acid frequencies of the chloroplast genomes of C. opulens. The squares below represent all the codons that encode each type of amino acid; the height of the column above represents the total sum of RSCU values for all codons; the height of each column represents the RSCU value for each codon. Fig. S5. Amino acid frequencies of the chloroplast genomes of C. jubata. The squares below represent all the codons that encode each type of amino acid; the height of the column above represents the total sum of RSCU values for all codons; the height of each column represents the RSCU value for each codon. Fig. S6. Amino acid frequencies of the chloroplast genomes of C. rosea. The squares below represent all the codons that encode each type of amino acid; the height of the column above represents the total sum of RSCU values for all codons; the height of each column represents the RSCU value for each codon. Fig. S7. Amino acid frequencies of the chloroplast genomes of C. microphylla. The squares below represent all the codons [file 12870_2024_4979_MOESM2_ESM.zip › Additional file 2/Fig. S9.png]
